# Clinical Outcomes of Oat Beta-Glucan Nutritional Intervention in Ulcerative Colitis: Case Reports of a Female and a Male Patient

**DOI:** 10.3390/nu17243812

**Published:** 2025-12-05

**Authors:** Alicja Zalecińska, Joanna Harasym, Katarzyna Dziendzikowska, Katarzyna Sikorska, Joanna Gromadzka-Ostrowska

**Affiliations:** 1 Doctoral School, Warsaw University of Life Sciences, 02-797 Warsaw, Poland; alicja_zalecinska@sggw.edu.pl; 2 Department of Biotechnology and Food Analysis, Faculty of Production Engineering, Wroclaw University of Economics and Business, 53-345 Wrocław, Poland; 3 Adaptive Food Systems Accelerator, Research Centre, Wroclaw University of Economics and Business, 53-345 Wrocław, Poland; 4 Department of Dietetics, Institute of Human Nutrition, Warsaw University of Life Sciences, 02-797 Warsaw, Poland; joanna_gromadzka-ostrowska@sggw.edu.pl; 5 Centre for Radiobiology and Biological Dosimetry, Institute of Nuclear Chemistry and Technology, 03-195 Warsaw, Poland; k.sikorska@ichtj.waw.pl

**Keywords:** calprotectin, dietary intervention, disease activity index, human ulcerative colitis, Mayo score, oat beta-glucan, 3D intestinal co-culture

## Abstract

**Background:** Inflammatory bowel diseases include Crohn’s disease (CD) and ulcerative colitis (UC). These diseases are characterized by periods of exacerbated inflammation of the gastrointestinal mucosa, interspersed with periods of remission. Current pharmacological interventions are only partially effective. There is a need for effective dietary therapies and interventions involving plant substances that can alleviate the course of this disease. **Objectives:** This study aimed to determine the effects of a 28-day dietary intervention involving a 3% solution of chemically pure, low-molar-mass oat beta-glucan (OBG) in patients diagnosed with de novo UC. Similar-aged men and women were compared. **Methods:** The OBG was isolated and prepared for consumption as a sterile aqueous suspension. This solution had previously been evaluated for in vitro toxicity using 3D intestinal co-cultures comprising Caco-2, HT29-MTX and THP-1 cells. Before and after the dietary intervention, endoscopic colon examinations were performed and blood hematological, biochemical and immunological parameters, as well as stool calprotectin concentrations, were analyzed. The Disease Activity Index (DAI), endoscopic Mayo score, the Lichtiger Colitis Activity Index (LCAI) and the neutrophil-to-lymphocyte ratio (NLR) were also determined. Following dietary intervention, the Mayo score, DAI, fecal calprotectin levels, and indices of peripheral blood white cells, CRP, and pro-inflammatory cytokine concentrations were decreased. **Results/Conclusions:** The obtained results demonstrated the beneficial effect of dietary intervention with OBG in accelerating the achievement of clinical remission in patients with UC.

## 1. Introduction

Inflammatory bowel disease (IBD), which encompasses Crohn’s disease (CD) and ulcerative colitis (UC), is a group of chronic disorders affecting the gastrointestinal tract. Both CD and UC are characterized by periods of exacerbation and remission [1]. UC is limited to the colonic mucosa and submucosa, with continuous inflammation that typically begins in the rectum and spreads proximally. Endoscopic findings include ulceration and inflammatory infiltrates, while clinical manifestations involve bloody diarrhea, abdominal pain, malnutrition, and weight loss [2]. This chronic condition significantly impairs patients’ quality of life. UC is driven by an enhanced immune response, which results in elevated levels of pro-inflammatory cytokines in blood plasma, including TNF-alpha, IL-1beta and IL-6 [3]. It occurs most frequently in Caucasian population, particularly in Europe and North America, with approximately 400 per 100,000 people affected in North America. In Europe, the annual incidence of UC is estimated at 10 new cases per 100,000 people. In Poland, the prevalence was 191 per 100,000 people in 2020, with higher values reported in men than in women [4]. UC typically affects young people, with peak incidence during the second decade of life.

The primary aim of UC treatment is to induce remission during the active phase, maintain the improvements achieved through induction therapy, and reduce the risk of relapse. Current pharmacological treatment, including aminosalicylates, corticosteroids, antibiotics and immunomodulators, is often limited in efficacy and does not result in complete remission. Complications such as perforations, fistulas, strictures and neoplastic changes worsen the prognosis for patients. The complications are difficult to treat, often require surgical or endoscopic interventions, and are associated with a high risk of recurrence, further diminishing patients’ quality of life [5]. In cases of mild to moderate UC limited to the rectum, treatment is initially administered locally. The first-line drug is 5-aminosalicylic acid (5-ASA, mesalazine), which induces remission in 31–80% of patients, compared to 7–11% in the placebo group. UC treatment pathways are divided into three main categories based on disease extent and severity: milder distal colitis, extensive colitis and severe disease. Each pathway follows a specific approach, intensifying therapy if the expected results are not achieved. The treatment options include 5-ASA, immunomodulators or biologic therapies. Topical corticosteroids, such as budesonide, are generally less effective than topically applied mesalazine [6,7]. In light of the limitations of conventional pharmacological approaches, there is growing interest in complementary strategies, including nutritional interventions, that may support disease management and improve patient outcomes.

Nutritional management in UC primarily aims to prevent malnutrition, which is a common complication caused by increased catabolism in patients with active, uncontrolled disease. Malnutrition is further exacerbated by nutrient deficiencies and side effects of pharmacotherapy [8]. Conversely, proper nutrition improves long-term outcomes, enhances the effectiveness of pharmacological treatments and supports surgical recovery. Diet is also considered an important environmental factor in the pathogenesis of IBD. Due to patients’ keen interest in the role of diet, a critical element of a holistic approach to caring for IBD patients is providing them with the opportunity to consult a qualified and experienced clinical dietitian. Dietary recommendations for patients with UC are individually formulated and typically include 5–6 small, regular, easily digestible meals daily [9,10]. Dietary fiber plays a significant role in UC management, with clinical benefits reported regardless of disease activity. Supplementation with selected types of dietary fiber may support remission and reduce mucosal damage over the course of the disease [11].

Oat beta-glucans (OBG), a form of soluble dietary fiber consisting of D-glucose molecules linked by β-1,3 and β-1,4 glycosidic bonds, demonstrate several biological activities, including prebiotic, cholesterol-lowering, anti-diabetic and immunomodulatory properties [12] OBG consumption also exhibits indirect antioxidant activity and modulate the expression of chemokines, their receptors and proteins associated with IBD [13,14]. Additionally, anti-carcinogenic properties of OBG have been demonstrated in animal models. In our recent study, dietary OBG supplementation significantly stimulated autophagy and apoptosis in the colonic mucosa during the early stages of carcinogenesis. This effect was dose-dependent, with a 3% OBG showing the most beneficial effect [15].

Therefore, the present study aims to evaluate the effects of a 28-day intervention using 100 mL of a 3% OBG solution, previously shown to be non-toxic, in patients with newly diagnosed UC. These findings may provide preliminary insights that could help future clinical studies on the role of dietary fiber, particularly OBG, in the management of UC.

## 2. Materials and Methods

### 2.1. Dietary Supplement

A low-molar-mass isolate of oat β-(1→3),(1→4)-D-glucan (OBG) was prepared from oat bran fiber sourced from Bestpharma (Warsaw, Poland) through a proprietary, patented extraction procedure previously documented in our earlier work [16,17]. The complete isolation and purification protocol for the beta-glucan is provided elsewhere [15]. Briefly, the oat bran underwent multiple freeze–milling cycles to reduce particle size, followed by alkaline extraction of beta-glucan at pH 8.5 using NaOH; the resulting supernatant was deproteinized at its isoelectric point. Enzymatic analysis performed with the Megazyme Kit (Megazyme by Neogen, Bray, Ireland) confirmed that the purified beta-glucan achieved 99.3% purity, resulting in low-weight white powder. For administration to patients, it needed to be prepared in a form that was safe and easy to swallow. In accordance with the EFSA recommendation, a 3% OBG solution was prepared by mixing the powder with ultrapure water. The solution was heated and stirred until homogeneous and then sterilized in an autoclave. Patients were advised to consume the OBG solution of total dosage of 3 g/per day.

### 2.2. In Vitro Evaluation of Oat Beta-Glucans on Cell Viability and Cytotoxicity

The human intestinal epithelial cell lines—Caco-2 and HT29-MTX cells—as well as THP-1 monocytes were obtained from the American Type Culture Collection (ATCC). A co-culture of Caco-2, HT29-MTX (Caco-2 cells: HT29 MTX in ratio 70%:30%—as in the large intestine) and THP-1 cells differentiated with phorbol-12-myristate 13-acetate (PMA) were grown on plate Corning^®^ HTS Transwell^®^-24 well permeable supports (Sigma Aldrich, St. Louis, MO, USA/CLS3398-2EA). These cells were maintained in a complete cell medium DMEM (Gibco, Waltham, MA, USA), containing 10% fetal bovine serum (FBS) (Gibco, Waltham, MA, USA). The 3D cell culture was maintained at 37 °C and 5% CO_2_.

The cytotoxicity of OBG was assessed by flow cytometry based on the fluorescence intensity of SYTOX™ Blue Dead Cell Stain (ThermoFisher Scientific, Waltham, MA, USA, S34857); the nucleic acids of dead cells fluoresce bright blue when excited with 405 nm violet laser light. Caco-2, HT29-MTX, and cells differentiated with PMA (Phorbol 12-myristate 13-acetate, Sigma-Aldrich), THP-1 cells, were seeded into 24-well 3D plate Corning^®^ HTS Transwell^®^ (Sigma Aldrich, St. Louis, MO, USA)-24 well permeable supports and grown overnight to reach 1.5 × 10^5^ cells/well on average (in a ratio 70%:30% Caco-2: HT29-MTX). The cells were incubated with OBG (100, 200 and 400 µg/mL) for 24 h. Subsequently, the cells were detached by trypsinization and spun down (200× *g*, 5 min); 1 µM of Sytox Blue was added and incubated for 5 min. The cells were washed once with the culture medium containing 10% FBS, resuspended in the culture medium containing 2% FBS, and analyzed by flow cytometry. Fluorescence was analyzed with a 440/40 nm filter using flow cytometry (LSRFortessa, BD Biosciences, Franklin Lakes, NJ, USA) (Supplementary Data, Table S3).

### 2.3. Study Design, Subjects, Clinical Symptoms and Main Complaint

This study was approved by the Rectoral Ethics Committee of Warsaw University of Life Sciences, Poland (Commission Decision No. 9/RKE/2024/U). Written informed consent for the study was obtained from both participants. The study was designed as a case report comparing clinical, endoscopic, hematological, and blood plasma biochemical indices in one woman and one man before and after a 28-day dietary intervention conducted at Warsaw University of Life Sciences and the Alicja Zalecińska Dietetic Clinic. The individuals met the predefined inclusion criteria: they were adults (aged ≥ 18 years) with a diagnosis of de novo exacerbation of UC, objectively confirmed by endoscopic assessment of the colon, supervised by a gastroenterologist. Each participant received pharmacological treatment and had individually scheduled consultations with a dietitian to establish dietary recommendations and provide nutritional education. Following enrollment, both patients began a 28-day dietary intervention, which included daily oral administration of 100 mL of a 3% solution of OBG, previously shown to be non-toxic in vitro. During the dietary intervention, both patients followed an easily digestible, anti-inflammatory diet using cooking techniques such as boiling, steaming, and braising. No side effects were observed during the dietary intervention period.

**Figure 1:**
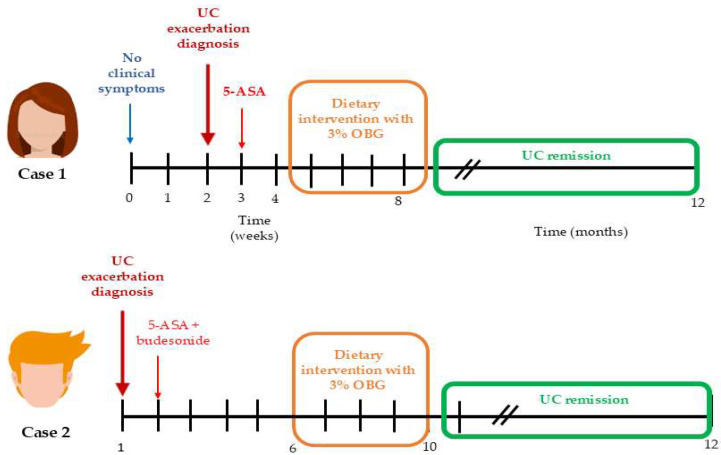
The case treatment timeline of each patient. Time scale in weeks in the initial phase, and in months after dietary intervention with 3% OBG.

The dietitian collected the participants’ medical histories consecutively during each visit, both before and after the dietary intervention. Clinical information was gathered at the pre-intervention visit, throughout the intervention period, and during the post-intervention visit. The parameters monitored included daily defecation frequency, presence of blood in the feces, abdominal pain and its severity, weight loss and appetite. One year after completing the study, both patients participated in an individual follow-up health interview conducted by the dietitian. Neither patient reported any history of illness or malaise prior to the exacerbation of UC. Body weight, hematochezia, gross rectal bleeding and stool consistency were assessed before and after dietary intervention. The Disease Activity Index (DAI) was calculated based on percent weight loss, presence of intestinal bleeding (no blood, occult blood, or gross blood) and stool consistency (normal, loose, or diarrheal) according to the following formula: DAI = body weight loss + diarrhea score + rectal bleeding score (Supplementary, Table S1) [18]. The Lichtiger Colitis Activity Index (LCAI) is a clinical tool used to assess the severity of symptoms in patients with ulcerative colitis. The scale interprets cut-off points as follows: 0–2: disease in remission (inactive), <10: response to treatment, ≥10: active disease and no response to treatment (Supplementary, Table S2) [19]. The case treatment timeline of each patient is provided in Figure 1.

### 2.4. Colonoscopy and Histopathological Examination

A rectosigmoidoscopy was performed in case 1 (female patient), whereas a colonoscopy was performed in case 2 (male patient). In both cases, tissue samples were collected for histopathological analysis. The endoscopic findings were assessed using the Mayo UC disease activity scale (MES). Histopathological analysis was performed to determine the presence of dysplasia, inflammatory infiltrates of mononuclear cells and neutrophil granulocytes, and cricoid and superficial ulcers. The gastroenterologist who performed the endoscopic examination and took a sample for histopathological analysis provided endoscopic images and a description of the mucous histology. However, no microscopic images were included.

### 2.5. Peripheral Blood Hematology and Biochemical Assays

Venous blood was collected from both participants (in cases 1 and 2) three days before and three days after the dietary intervention. Sampling was performed in the morning between 08:00 and 10:00 after a 12-h overnight fast. A certified medical laboratory performed analyses of whole blood hematology, serum biochemistry liver enzyme activity (alanine and aspartate aminotransferases), C-reactive protein (CRP) and cytokine concentrations (IL-1beta, IL-8, IL-10, IL-12 and TNF-alpha). Hematological parameters were analyzed by flow cytometry, spectrophotometry and impedance using a Sysmex XN-10TM hematology analyser (Sysmex Company, Kobe, Japan). Serum CRP levels were determined using an immunoturbidimetric method on an ABBOTT ALINITY C analyser (Abbott Laboratorie Company, North Chicago, IL, USA). Serum AST and ALT activities were analyzed using a spectrophotometric method (Cobas 6000 apparatus, Roche Diagnostics, Vienna, Austria). Cytokine concentrations in serum (IL-1beta, IL-8, IL-10, IL-12 and TNF-α) were analyzed using a chemiluminescent immunoassay. The neutrophil-to-lymphocyte ratio (NLR) was calculated from the hematological parameters as an additional marker of systemic inflammation.

### 2.6. Calprotectin and Occult Blood Stool Assays

The fecal calprotectin (FC) concentration was measured in a certified medical laboratory using an immunoenzymatic ELISA Assay (EUROIMMUN Analyser I, Wrocław, Poland). A fecal occult blood test was also performed in the same laboratory.

### 2.7. Statistical Analysis

For the in vitro experiment, statistical differences among means were determined by two-way analysis of variance, *p* < 0.05, accompanied by the Bonferroni test. All calculations were made using GraphPad Prism 5.0 software (GraphPad Software, Inc., San Diego, CA, USA). Results were presented as a mean of three experiments ± SD.

## 3. Results

### 3.1. The In Vitro Toxicity of Beta-Glucan

**Figure 2:**
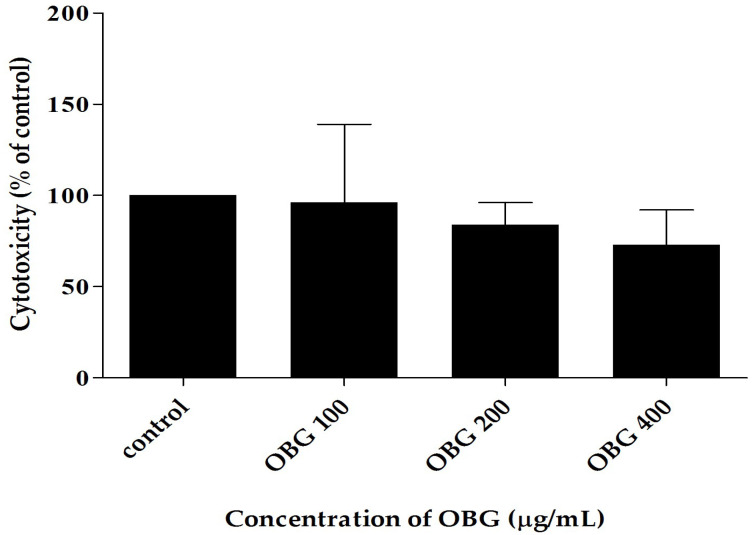
The toxicity of oat beta-glucan estimated by flow cytometry in 3D intestine co-culture.

After a 24-h incubation period, regardless of the concentration of these polysaccharides in the medium, no statistically significant toxicity of oat beta-glucan was observed in the 3D intestinal co-culture (Caco-2, HT-29-MTX, and THP-1 cells) (Figure 2).

### 3.2. The Clinical Characterization, Symptoms and Disease Activity Index (DAI)

Case 1: a woman.

A 27-year-old non-smoking Caucasian woman was diagnosed with UC based on endoscopic evaluation (a rectosigmoidoscopy) and clinical symptoms, including abdominal pain and an average of 15 stool frequency per day. Stool form was assessed using the Bristol Stool Form Scale (BSFS), with types from 5 to 7, indicating diarrhea ranging from mild to very watery with mucus. She was treated 3 weeks before dietary intervention with mesalazine, the preferred drug for inducing and maintaining remission of mild to moderate UC.

Case 2: a man.

A 20-year-old non-smoking Caucasian man was diagnosed with active UC following colonoscopic evaluation. The patient reported symptoms including rectal bleeding and severe abdominal pain in the two months prior to diagnosis, with up to 20 stools per day. According to the BSFS, his stools were categorized as types 6–7, indicating severe diarrhea. Pharmacological treatment was initiated 6 weeks before dietary intervention, including mesalazine and budesonide, a corticosteroid used to induce remission in UC. The severity of disease in case 2 was much greater than in case 1. Table 1 presents a comparative clinical summary of both cases.

After the 28-day dietary intervention, both patients (cases 1 and 2) experienced a reduction in diarrhea and a decrease in stool frequency, with the stool becoming more formed. In case 2, rectal bleeding, previously reported as a worsening symptom, stopped completely. Case 1 showed stabilization of body weight, increasing from 54 kg to 57 kg, along with improved appetite. Case 2 also demonstrated a slight increase in body weight (from 84 to 86 kg). Neither patient reported abdominal pain at the end of the intervention. The DAI was calculated before and after the dietary intervention to assess the severity of UC. After 28 days, the DAI score decreased from 5 to 0 in the female patient and from 8 to 1 in the male patient, indicating disease remission. The DAI score was confirmed by an assessment using the Lichtiger Colitis Activity Index (LCAI), a clinical tool that assesses symptom severity in patients with UC. The LCAI decreased from 16 to 1 in a woman and from 19 to 2 in a man, indicating that remission occurred in both cases following dietary intervention.

One year after the study ended, both patients remained in clinical remission. They reported no abdominal pain, no blood in the stool, and a maximum of two bowel movements per day, with fully formed stools.

### 3.3. Endoscopy and Histological Examination of Biopsy Specimens

**Figure 3:**
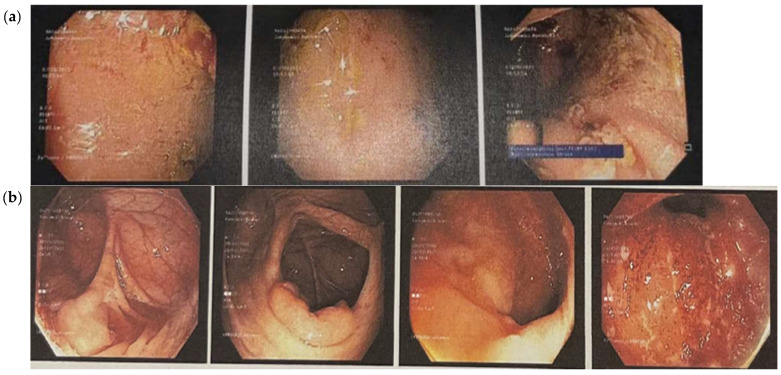
Endoscopic images showing the state of the bowel before the dietary intervention, indicating a diagnosis of ulcerative colitis. (**a**) Case 1: Rectosigmoidoscopy images showing mucosal swelling, marked erythema and loss of vascular pattern. There is evidence of contact bleeding and presence of erosions. (**b**) Case 2: colonoscopic images showing an obliterated vascular pattern that is granulated and dull, with numerous aphthae, ulcers and evidence of contact bleeding.

For Case 1, rectosigmoidoscopy assessment revealed features consistent with moderately active disease, corresponding to a MES of 2. This indicates significant mucosal inflammation without spontaneous hemorrhage or frank ulceration (Figure 3a). Clinically, the patient had reported a three-month history of rectal bleeding.

For Case 2, colonoscopic evaluation revealed severely active disease, with a MES of 3. The key features included spontaneous mucosal bleeding and the presence of ulceration, indicating a high degree of inflammatory activity (Figure 3b). The microscopic evaluation of rectal mucosal tissue confirmed active inflammation with crypt abscess formation and the presence of aphthous erosions.

**Figure 4:**
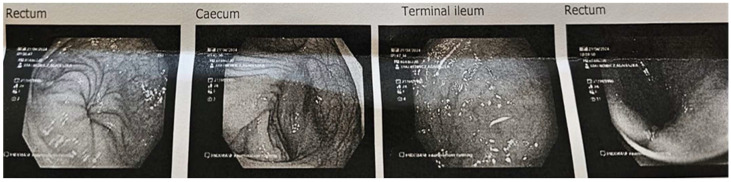
Case 1, 28 days after OBG dietary intervention: colonoscopy images of the bowel and ileum, which appeared normal. Very mild erythema was noted on the rectum.

After the 28-day dietary intervention with 3% OBG, a follow-up colonoscopic examination was performed in case 1. The procedure revealed a clear reduction in mucosal inflammation compared to baseline findings (Figure 4). The gastroenterologist decided not to repeat the colonoscopic examination in case 2 due to the risk of inducing contact bleeding.

### 3.4. Peripheral Blood Hematology

Before dietary intervention, both patients had lower red blood cell indices, including erythrocyte count, hemoglobin concentration and hematocrit values, compared to values observed after 28 days of OBG supplementation. However, all changes were within the referenced range. Leukocyte count was also lower before the dietary intervention, with case 1 already showing signs of leukopenia. In both cases, this parameter increased after the dietary OBG intervention. In case 1, the lymphocyte count was higher before the dietary treatment and decreased slightly afterwards; in case 2, this parameter remained unchanged. Platelet count decreased in case 1 and remained stable in case 2. Neutrophil counts were within the normal range in both cases before the dietary intervention and increased in both cases after the dietary intervention. The NRL index was calculated, and, in both cases, values were lower before the dietary intervention than after it (Table 2).

### 3.5. Blood Serum Biochemical and Immunological Parameters Before and After OBG Dietary Intervention

Serum activities of ALT and AST were lower before the dietary intervention than after 28 days of OBG consumption, in both women and men. However, enzyme activity levels remained within normal reference ranges throughout the study. The blood serum CRP levels in case 1 were similar before and after the intervention, with both values within the normal range. In contrast, a significant decrease in serum CRP concentration was observed after the dietary intervention in case 2. It should be noted that the CRP concentration in case 2 before the intervention was very high, exceeding the upper limit of the normal range (9.4 mg/L versus 0.0–5.0 mg/L).

After the intervention, the case 2 CRP concentration decreased to less than 1 mg/L. The serum Il-1beta concentration remained unchanged in case 1 before and after the intervention, while it decreased significantly in case 2 after the OBG intervention. The Il-6 concentration in case 1 was above normal, increasing markedly after the intervention. In case 2, IL-6 concentrations were within the normal range before and after the intervention, although a slight increase was observed after the OBG intervention. In case 1, the IL-8 concentration was above normal before intervention and decreased after treatment. In case 2, the blood serum IL-8 concentration was within the normal range before and after the OBG intervention. Similarly, the IL-10 concentration in both patients was within the normal range before and after the dietary intervention.

Both cases showed an increase in IL-10 after OBG treatment. IL-12 concentrations were also within the normal range before and after the OBG intervention in both cases, with a slight increase after OBG treatment and lower values in case 1 than in case 2 before intervention. TNF-alpha concentrations were within the normal range in both cases before and after the OBG intervention. In case 1, concentrations of this cytokine decreased after the intervention, while in case 2 they increased (Table 3).

### 3.6. The Fecal Calprotectin Level and Presence of Occult Blood

In both cases, the stool calprotectin level was significantly above the normal range (>51 μg/g) prior to dietary intervention. In case 1, the result was six times higher than the upper limit of the referenced range (316.0 μg/g), and in case 2, it was almost fifty-three times higher (2640.0 μg/g). After 28 days dietary intervention this parameter was <5.0 and 1.0 μg/g, respectively. Fecal occult blood was observed only in case 2 before dietary intervention. In both cases, the presence of fecal occult blood was not observed following the dietary intervention.

## 4. Discussion

Our study is the first case study in which patients with ulcerative colitis (UC) in the acute phase of de novo disease underwent a 28-day nutritional intervention with low-molecular-weight oat beta-glucan (OBG) at a dose of 3 g/day. Prior to its use as a dietary supplement in patients with UC, this preparation was evaluated for cytotoxicity in vitro using a 3D model of the human colon wall cells. Notably, both the in vitro cytotoxicity evaluation and the present clinical intervention were conducted using the same low molecular weight OBG preparation, which allows for a coherent interpretation of its safety profile and biological activity across experimental models. This preparation has also been extensively evaluated by our team in animal in vivo studies using various models of gastrointestinal disorders, further supporting its biocompatibility and functional relevance.

After 24 h of incubation, no statistically significant toxicity of OBG was observed in the 3D intestine co-culture comprising Caco-2, HT29-MTX and THP-1 cells. Our results are compatible with Suraiya et al. [20], who showed that the beta-glucan extracted from some fungi exhibited no cytotoxic effects in Caco-2 cell culture. In that study, cell viability was only slightly reduced by beta-glucan extracted from *M. purpureus* and *M. kaoliang* (to 92.10% and 89.24%, respectively) and to 90.68% by yeast-derived beta-glucan (*S. cereviacese*). These observations are consistent with the broader understanding of beta-glucan biocompatibility, although its biological effects may differ depending on structural characteristics such as molar mass. Some studies have suggested that the molar mass of beta-glucan may also influence its cytotoxicity effect; however, Choromanska et al. [21] found that high-molar-mass OBG showed no toxicity toward human keratinocytes (HaCaT cells). In general, beta-glucans are considered safe bioactive compounds suitable for use as dietary supplements [22]. Interestingly, while they are non-toxic to healthy cells, several studies have demonstrated selective cytotoxicity of beta-glucans toward cancer cells, indicating potential antitumor properties. For instance, Upadhyay et al. [23] demonstrated that yeast-derived beta-glucan (50–150 μg/mL) induced apoptosis in HeLa cervical cancer cells by promoting ROS generation, DNA fragmentation, and reduced mitochondrial membrane potential. Similarly, Al-Khuzaay et al. [24] reported concentration- and time-dependent cytotoxicity of *Euglena gracilis* beta-glucan on MCF-7 and AMJ13 breast cancer cells, with significant effects at higher concentrations (500–1000 µg/mL, 72 h). Additionally, Boulifa et al. [25] showed that yeast beta-(1→3)(1→6)-D-glucan inhibited breast cancer proliferation in 2D models at 100 μg/mL, with the strongest effects on MCF-7 cells, although no significant effects were seen in 3D tumor spheroids. The impact of molar mass on cytotoxicity was further confirmed by Choromanska et al. [21], who demonstrated that high-molar-mass OBG reduced the viability of A549 and H69AR lung cancer cells by 60–70% (at 200 μg/mL) accompanied by increased oxidative stress. In contrast, low-molar-mass OBG induced milder effects with increased MnSOD expression, suggesting a different mechanism of action depending on molecular weight.

The selective effects of beta-glucans on different cell types can be explained through their role as pathogen-associated molecular patterns (PAMPs) and biological response modifiers (BRMs). Based on preclinical studies, we hypothesize that beta-glucans engage pattern recognition receptors (Dectin-1, CR3, scavenger receptors) on innate immune cells, initiating signaling cascades that enhance phagocytosis and cytokine production [26,27,28,29]. Crucially, beta-glucans appear to exert context-dependent effects: pro-inflammatory responses against pathogens and malignant cells, but anti-inflammatory and protective effects in normal tissues through activation of antioxidant pathways [30,31,32]. This dual action may create a therapeutic window relevant to UC management, though these mechanisms require validation in controlled human studies.

For both patients, who were young people aged 20 and 27, the medical history did not reveal any other conditions apart from UC, which had relapsed, prompting both patients to visit a gastroenterologist and a dietitian. As shown by examinations of both the clinical condition of the patients and changes in the biochemical parameters of peripheral blood and stool, as well as endoscopic examination of the intestine, after 28 days of using OBG, many of these indicators normalized spectacularly. This included the clinical symptoms experienced by the woman and man, such as the frequency of bowel movements during the day, which decreased several times, significantly improving their quality of life, especially as this was accompanied by the disappearance of pain. A measurable indicator of disease remission was a decrease in the DAI and LCAI clinical indices, and above all, a significant reduction in fecal calprotectin (FC) and C-reactive protein (CRP) levels in peripheral blood serum.

The disease activity index (DAI) in UC is a standardized tool for the objective assessment of disease severity, often used primarily in clinical practice and based on four parameters that do not require any laboratory tests: frequency of bowel movements, rectal bleeding, abdominal pain, and general well-being. The DAI is also used in scientific research conducted on animal models [33]. Another indicator of UC activity is the Lichter’s Clinical Activity Index (LCAI), based on eight clinical variables, also requiring no additional laboratory tests [19], and both indices are very good at determining the activity of the disease. As we found in our study, after 28 days of nutritional intervention with OBG, the values of both indices in both women and men decreased significantly, indicating the achievement of an inactive form of the disease or remission.

FC remains the closest to the “ideal” marker of intestinal mucosal healing in clinical practice [34]. Studies by other authors have shown that FC levels are significantly correlated with both the endoscopic stage and extent of mucosal lesions in patients with UC and the severity of clinical symptoms. Therefore, this easily obtainable and measurable marker is very useful for the precise assessment of the condition of the mucosa [35,36]. It should be added that FC levels are significantly correlated with clinical disease activity indices, endoscopic indices, and serum inflammation biomarkers in patients with UC, giving it high predictive value for assessing mucosal healing in these individuals [37]. In addition, FC levels correlate better with endoscopically confirmed disease activity than clinical examinations or CRP and hemoglobin concentrations or platelet and leukocyte counts in the blood. Such a strong correlation with endoscopic UC diagnosis suggests that FC is a useful biomarker for non-invasive monitoring of disease activity in patients [19,38].

Following this line of reasoning, FC measurement may enable the use of targeted treatment to prevent UC recurrence or the development of new therapeutic strategies to maintain symptomatic remission [39]. As demonstrated in studies by other authors, high FC levels were associated with high MES values and severe histopathological changes, which is consistent with the above-cited reports and emphasizes the importance of FC as a very good biomarker of intestinal inflammation in IBD [40]. It is accepted that FC levels above 250 μg/g indicate very severe inflammation in the intestine, and our patients had FC levels of 316 μg/g (female) and even 2640 μg/g (male), which in the latter case is 53 times higher than the maximum level of this protein considered physiological, indicating a more active form of the disease. The upper limit of the physiological norm is 50 μg/g. In both cases, dietary intervention with 100 mL of 3% OBG/day for 28 days in combination with pharmacological treatment resulted in a significant reduction in this parameter below the normal range, which in turn indicates complete healing of the intestinal mucosa in both patients.

As stated in other studies, low FC levels correlate well with histological remission and mucosal healing in UC located in the colon [41]. It should be added that even one year after the end of the OBG intervention, the clinical symptoms in both patients, including general well-being, weight stabilization, and frequency of bowel movements, did not indicate a recurrence of the disease. The concentration of C-reactive protein (CRP) in the blood, alongside FC, is also considered a good biomarker of inflammation, especially in patients with UC. C-reactive protein is produced by the liver in response to an increase in the secretion of pro-inflammatory cytokines (IL-1beta, IL-6, TNF-alpha) [34] and is an effective indicator for assessing the activity of this disease, especially in combination with other parameters such as the number of bowel movements, the presence of blood in the stool, body temperature, and blood count parameters [42].

In UC, elevated serum CRP levels indicate active inflammation and are associated with a more severe course of the disease. This concentration correlates with clinical and histological disease activity and is used to monitor treatment efficacy. A meta-analysis of the accuracy of CRP in the diagnosis of IBD reported a mean sensitivity and specificity of 66% and 82% for UC and 79.5% and 61% for CD, respectively. An analysis of the use of CRP as a marker of intestinal mucosal healing showed that higher sensitivity than specificity was characteristic of CD, with median values of 79.5% compared to 61%, while greater specificity than sensitivity characterized UC, with median values of 82% compared to 66% [34]. A reduction in CRP concentration suggests a positive response to therapy, which was the case in the male patient, as persistently high CRP concentrations may indicate treatment failure [43]. In the case of the female patient, the exacerbation of the disease did not cause an increase in CRP concentration, and the differences between the measurements before and after the intervention with OBG were minor. In the case of the man, whose exacerbation of the disease was assessed on the Mayo scale as severe (MES = 3), the CRP concentration before the intervention was almost twice as high as the upper limit of the normal range, reaching this normal range after 28 days of using the aforementioned intervention.

The Mayo Endoscopic Score (MES) is a 4-point scale (0–3) used to assess the severity of inflammation in UC, based on features such as erythema, vascular patterns, friability, erosions, ulcers, and bleeding diagnosed during endoscopy. A score of 0 indicates a normal condition, 1 indicates mild disease, 2 indicates moderate disease with erosions, and 3 indicates severe disease with ulcerations and spontaneous bleeding. The MES is widely used in clinical trials and clinical practice to assess disease activity and make treatment decisions [44]. The guidelines of the European Crohn’s and Colitis Organization and the Japanese Gastroenterological Association limit total endoscopic remission (normal or completely healed mucosa) to a score of 0 [45,46]. Since mucosal healing is a key therapeutic goal in the treatment of IBD, and objective assessment of the condition of this intestinal structure is paramount, endoscopic examination of the intestine remains the gold standard. In our study, rectosigmoidoscopy in women before OBG intervention showed results corresponding to MES = 2, indicating significant inflammation of the intestinal mucosa, while colonoscopy in men showed a severe form of the disease, corresponding to MES = 3, characterized by spontaneous bleeding of the mucosa and the presence of ulcers, which indicated a high degree of inflammatory activity. In the woman, after 28 days of intervention with OBG, MES was reduced to 0, indicating healing of the intestinal mucosa.

In this patient’s case, the combination of strict dietary recommendations and adequate hydration by drinking at least 2 L of water per day (which the man did not follow) and pharmacological treatment resulted in a spectacular reduction in colon inflammation to such an extent that the attending physician decided to perform a repeat colonoscopy, which indicated clinical remission confirmed by the normalization of other parameters, including the elimination of clinical symptoms and a significant reduction in FC and CRP. The activity of liver transaminases in the blood serum of both patients was not elevated either before or after 28 days of intervention, indicating no liver damage that could be the result of, for example, the medications used. The first-line drug mesalazine, which is considered safe based on current knowledge [47], shows low hepatotoxicity due to minimal absorption and mostly excretion in the feces [48].

The concentration of cytokines, which are protein signaling molecules produced by activated immune cells during all inflammatory processes, including those related to the etiology and development of IBD, varies significantly during the development of inflammation. Among cytokines, interleukins are crucial for the development and severity of UC, with some causing inflammation and their elevated levels contributing to the chronic immune-mediated inflammation characteristic of UC, while others inhibit the development of inflammation in the intestinal mucosa. In people with UC, changes in interleukin levels primarily affect the intestinal mucosa, while changes in the concentration of these cytokines in peripheral blood serum are not as pronounced and do not correlate well with the clinical activity of UC [43]. This was probably the reason why, in both patients, the changes observed before and after the nutritional intervention were minor, and the serum concentration of most of the analyzed interleukins did not exceed the norm. The exception was the changes observed in Case 2, where the concentration of IL-1beta was reduced more than threefold after the intervention. This was accompanied by an increase in the concentration of anti-inflammatory IL-10 after 28 days of intervention with OBG. In contrast, the concentration of IL-6 in the woman was higher than normal both before and after the intervention, with a higher value after the intervention. The results of a large prospective study showed that serum IL-6 concentration is associated with disease activity in both CD and UC, with higher concentrations occurring in the active form of the disease [49]. The differences between our results and those of this study were probably due to the high individual variability characterizing this parameter. It should be added that therapies aimed at inhibiting the action of interleukins, in particular by blocking IL-12/23 and IL-1beta receptors with various compounds, are a key element of modern medical strategies for treating UC [50].

Similarly, red blood cell parameters, as well as leukocytes and their subpopulations, did not change significantly in the man when comparing the values before and after the intervention, and all were within the normal range of the laboratory performing the analysis. However, it should be added that the only changes outside the laboratory norm were found in the woman and concerned the number of leukocytes, neutrophils, and the NLR index, all of which were significantly below normal before the intervention and reached normal levels after the OBG intervention.

In UC, hematological parameters often change, the most common being anemia manifested by low hemoglobin concentration, which is most often caused by significant blood loss due to massive bleeding. Other indicators of disease activity include increased white blood cell, neutrophil, and platelet counts, as well as changes in the neutrophil-to-lymphocyte ratio (NLR). Hematological abnormalities can also be observed in the preclinical phase of the disease, potentially many years before diagnosis [51]. Celikbilek and his team showed that NLR is a marker associated with the severity of inflammation also in UC, and Ma and his team proved that the best cutoff point for NLR is 1.91–3.10, which is exactly the same as the normal range in our study. It should be added that the use of drugs may affect the NLR value [52,53]. On the other hand, considering the results of large clinical trials, no association between disease activity parameters and any of the calculated blood indices, such as NLR, has been demonstrated in either CD or UC patients. Based on these results, it can be concluded that inflammation indicators based on blood test results cannot be used as surrogate biomarkers of disease activity in IBD [54].

One of the limiting factors in our study is the fact that it was performed on only two individuals, and in one of them, due to the possibility of massive bleeding, the physician decided not to repeat the endoscopic examination after the intervention. The conclusions are also limited by the fact that the laboratory where the endoscopic examination was performed did not provide the results and images of the histopathological examination, so the conclusion that the inflammation-damaged intestinal mucosa had healed was based on the assessment of other biochemical and clinical parameters. The strengths of this study are the consistent correlation between FC and endoscopic, clinical, and biochemical assessments, indicating the effectiveness of nutritional therapy with OBG.

Our study is the first to use a dietary intervention with a highly purified 3% low molecular weight oat beta-glucan for 28 days in combination with pharmacotherapy and nutritional recommendations. The aim of the dietary intervention with OBG was to support pharmacological treatment and induce clinical remission. Both the woman and the man achieved clinical remission, as indicated by UC-specific parameters such as fecal calprotectin and CRP concentration, and in the case of the woman, remission was also confirmed by endoscopy. Wider application of the above-described dietary intervention requires further research, but the results of this study indicate very beneficial effects in combination therapy with pharmacotherapy and may represent a new direction of support in the treatment of UC. It should be emphasized that OBG dietary supplementation did not adversely affect the well-being of patients and did not exacerbate clinical symptoms, which proves the safety of this preparation.

Moreover, Case 1 received mesalazine (3 g/day) for one week prior to OBG supplementation, while Case 2 received mesalazine plus budesonide (9 mg/day) for six weeks before OBG introduction (Figure 1). Despite these treatment durations, both patients exhibited LCAI scores indicating no response to treatment (LCAI = 16 and LCAI = 19). Following 28 days of OBG intervention, LCAI scores decreased to 1 and 2, indicating remission. This temporal relationship suggests OBG altered the intestinal environment to enable conventional pharmacological agents to function effectively. At baseline, FC concentrations of 316 μg/g (Case 1) and 2640 μg/g (Case 2) indicated massive neutrophil infiltration, while serum CRP of 9.4 mg/L (Case 2) reflected systemic inflammation. Elevated IL-6 (Case 1) and paradoxically low NLR (0.9–1.1) in both cases suggested dysregulated immune activation persisting despite pharmacotherapy. Due to pharmacological treatment, clear causal relationships between oat beta-glucan and UC remission cannot be established. However, temporal association between the dietary intervention and symptom resolution—particularly in the context of previously ineffective pharmacotherapy—suggests a potential adjunctive role of OBG in UC management. This temporal relationship between OBG supplementation and improvement in clinical, inflammatory, and immunological parameters suggests that OBG may have contributed to restoring gut homeostasis and thereby enhanced the effectiveness of ongoing pharmacotherapy.

It is important to note that while mesalazine acts primarily through inhibition of NF-κB, it does not directly target key mechanisms such as epithelial barrier integrity, gut microbiota dysbiosis, or metabolic reprogramming of immune cells. Similarly, budesonide offers localized immunosuppression but lacks the ability to modulate microbial composition or improve epithelial tight junction function. The rapid clinical improvement following OBG supplementation, particularly after previously ineffective pharmacotherapy, raises the possibility that OBG played a supportive role in modulating the intestinal environment—through its prebiotic, anti-inflammatory, and barrier-protective effects—and thereby enabled or enhanced the action of conventional drugs.

While the precise mechanisms underlying the observed clinical improvements cannot be definitively established from this case report, preclinical evidence suggests potential pathways by which OBG may have contributed to therapeutic response. Based on animal and in vitro studies, we hypothesize that OBG may have (1) engaged pattern recognition receptors (Dectin-1, CR3) on innate immune cells, promoting balanced immune responses and reprogramming from pro-inflammatory to tissue-reparative phenotypes; (2) undergone colonic fermentation to produce short-chain fatty acids (butyrate, propionate, acetate) that support colonocyte function, strengthen barrier integrity, and exert anti-inflammatory effects; and (3) facilitated metabolic shifts in immune cells through activation of the pentose phosphate pathway and enhanced antioxidant defenses. The observed reductions in calprotectin (316 to <5 µg/g; 2640 to 1 µg/g), normalization of inflammatory markers (CRP, IL-1β), increase in IL-10, and restoration of clinical parameters are consistent with these proposed mechanisms but require validation in controlled clinical trials with mechanistic endpoints.

Both patients adhered to the dietary protocol and tolerated the OBG supplementation well throughout the 28-day intervention. No adverse effects, such as gastrointestinal discomfort, bloating, skin reactions, or abdominal pain, were reported during or after the intervention period. Stool frequency and consistency improved steadily, and no exacerbation of symptoms occurred. Dietary recommendations for patients with UC vary depending on the severity of the disease. Current guidelines apply to periods of exacerbation and specify not only food groups to be excluded, but also recommendations on how to prepare meals, heat treatment, and the introduction of high-protein and high-energy supplements known as ONS (Oral Nutritional Supplements). Current knowledge confirms that diet has an impact on the pathogenesis of UC. A properly composed diet reduces the severity of clinical symptoms and improves patients’ well-being, nutritional status and, ultimately, response to pharmacological treatment. For this reason, researchers are looking for new dietetic solutions to support pharmacological treatment by designing new nutritional interventions, such as the pilot study designed by Narimani [55,56]. The general dietary recommendations introduced for our both patients were consistent with these guidelines. Additional beta-glucan supplementation had a synergistic effect.

The female patient followed the dietary recommendations closely, including the prescribed food preparation methods and daily OBG intake. The male patient also showed good compliance, although some day-to-day variability in dietary adherence was observed, which is common in clinical practice. Nevertheless, both patients implemented sustained dietary changes following the nutrition education provided. From a clinical standpoint, the intervention appeared safe and well tolerated. However, as a precautionary note, the use of viscous dietary fibers such as OBG may warrant caution in patients with intestinal strictures or severe active diarrhea. These factors should be considered when tailoring individual nutritional strategies in ulcerative colitis.

Comparison with previous human studies reveals important distinctions in our approach. Most existing research on β-glucan supplementation in inflammatory bowel disease has focused on oat bran or mixed fiber formulations in patients with quiescent disease or in the context of general dietary fiber interventions [55,56]. The present study differs in several critical aspects: (1) we used highly purified (99.3%), low-molecular-weight oat β-glucan rather than whole oat bran, eliminating confounding effects from other bioactive compounds; (2) the intervention targeted patients with active, de novo UC rather than maintenance therapy in stable disease; (3) both patients had previously shown inadequate response to standard pharmacotherapy (LCAI scores of 16 and 19 despite 3–6 weeks of mesalazine ± budesonide), suggesting OBG may address therapeutic gaps in conventional management; (4) the specific dosage (3 g/day) and delivery format (sterile aqueous solution) were designed for optimal tolerance during acute inflammation. These distinctive features—particularly the use of purified β-glucan as adjunctive therapy in pharmacotherapy-refractory active disease—represent a novel approach that warrants further investigation in controlled clinical trials to determine whether this intervention can be generalized beyond these preliminary observations.

## 5. Conclusions

In vitro studies showed no OBG cytotoxicity on intestinal co-cultures, indicating beta-glucans stimulate immune cells while protecting epithelial integrity. This context-dependent action—pro-inflammatory against pathogens but anti-inflammatory during barrier restoration—appears critical for therapeutic efficacy.

The temporal relationship is particularly informative in Case 2, where six weeks of dual pharmacotherapy failed to induce remission (LCAI = 19), yet OBG addition for 28 days achieved near-complete remission (LCAI = 2). This suggests OBG created conditions enabling pharmacological function by restoring barrier integrity (reducing antigenic stimulation), modulating the microbiome (increasing SCFAs), and reprogramming innate immunity (shifting from destruction toward repair). Sustained remission one-year post-intervention in both patients suggests fundamental restoration of intestinal homeostasis rather than symptom suppression.

These findings indicate dietary intervention with purified low-molecular-weight oat beta-glucan may represent an adjunctive strategy for UC patients with inadequate pharmacotherapy response, addressing barrier dysfunction, dysbiosis, and immune dysregulation to create conditions conducive to drug efficacy and mucosal healing.

## Figures and Tables

**Table 1:** A comparative clinical summary of both cases before dietary intervention.

	Case 1 (A Woman)	Case 2 (A Man)
Age (years)	27	20
Diagnosis	Ulcerative Colitis	Ulcerative Colitis
Mayo Endoscopic score (MES)	2 (Moderate)	3 (Severe)
Key Endoscopic Findings	Edema, erythema, absent vascular pattern, friability/contact bleeding, erosions	Spontaneous bleeding, ulceration
Stool Frequency (per day)	~15	Up to 20
The disease activity index (DAI)	5 (moderate active disease)	8 (active disease course)
Lichtiger Colitis Activity Index (LCAI)	16 (An active disease course and no response to treatment)	19 (An active disease course and no response to treatment)

**Table 2:** Peripheral blood hematological indices before and after OBG dietary intervention.

Parameter	Case 1 Before/After	Case 2 Before/After	Reference Range
RBC × 10^6^/μL	4.32/4.75	5.3/5.57	4.3–5.8
Hemoglobin [g/dL]	12.2/13.0	14.6/16.1	12.0–18.0
Hematocrit [%]	37.2/39.4	45.6/48.0	34.0–52.0
WBC × 10^3^/μL	3.33 /4.4	5.3/6.49	4.2–10.0
Lymphocytes × 10^3^/μL	1.47/1.2	2.23/2.25	1.3–4.0
Neutrophils × 10^3^/μL	1.46/2.78	2.43/3.54	1.78–6.04
Platelets × 10^3^/μL	225/196	296/292	140.0–450.0
NLR	0.9/2.3	1.1/1.6	1.91–3.10

**Table 3:** Blood serum parameters of cases 1 and 2 before and after OBG dietary intervention.

Parameter	Case 1 Before/After OBG Supplementation	Case 2 Before/After OBG Supplementation	Reference Range
ALT [u/L]	18 /25	12.9/44	0.0–50
AST [u/L]	44.0/53.0	19.9/37.0	0.0–130
CRP [mg/L]	1/<1	9.4/0.8	0.0–5.0
Il-1beta [pg/mL]	1.8/1.8	<6.7/<2.0	<6.7
Il-6 [pg/mL]	2.3/2.9	1.4/1.8	<2.0
Il-8 [pg/mL]	3.3/2.8	1.5/<3.0	<3.0
Il-10 [pg/mL]	1.2/1.6	1.9/<2.8	<2.8
Il-12 [pg/mL]	1.5/<1.9	1.1/<1.9	<1.9
TNF-alpha [pg/mL]	<3.7/2.4	4.1/<7.2	<7.2

## Data Availability

The original contributions presented in this study are included in the article/Supplementary Material. Further inquiries can be directed to the corresponding author.

## Supplementary Materials

The following supporting information can be downloaded at: https://www.mdpi.com/article/10.3390/nu17243812/s1: Table S1. Disease activity index (DAI) score; Table S2. The Lichtiger Colitis Activity Index (LCAI) before and after dietary intervention; Table S3. In vitro toxicity OBG data.

## Abbreviations

The following abbreviations are used in this manuscript:

IBD	inflammatory bowel disease
CD	Crohn disease
UC	ulcerative colitis
DAI	Disease Activity Index
LCAI	Lichtinger Colitis Activity Index
MES	Endoscopic Mayo score
BSFS	Bristol Stool Form Scale
OBG	low-molar-mass oat beta-glucan
FC	fecal calprotectin
CRP	C-reactive protein
ALT	alanine aminotransferase
AST	aspartate aminotransferase
WBC	white blood cells
RBC	red blood cells
NLR	neutrophil-to-lymphocyte ratio
5-ASA	5-aminosalicylic acid
PAMP	pathogen-associated molecular pattern
BRM	biological response modifier
ROS	Reactive oxygen species
TNF-alpha	tumor necrosis factor-alpha
IL-1beta	interleukin-1 beta
Il-6	interleukin-6
Il-8	interleukin-8
Il-10	interleukin-10
Il-12	interleukin-12
CR3	complement receptor 3
NK	natural killer cells
NF-kB	nuclear factor kappa-B
THP-1	Tohoku Hospital Pediatrics-1 cells line
HT29-MTX	human colon cancer cell line Methotrexate resistant
HeLa	immortal cell lines originating from Henrietta Lacks’ cervical cancer
