# Supplementary material for: Clinical Outcomes of Oat Beta-Glucan Nutritional Intervention in Ulcerative Colitis: Case Reports of a Female and a Male Patient
Source: Nutrients. 2025 Dec 5;17(24):3812. doi: 10.3390/nu17243812 (PMC12735812; doi:10.3390/nu17243812)
Supplement: Supplementary file 1 [file nutrients-17-03812-s001.zip › nutrients-3950198-supplementary.pdf]

Supplementary Data

Table S1. Disease activity index (DAI) score

Score	Body weight decrease (%)	Stool consistency	Rectal Bleeding
0	<1	Normal	Normal
1	1-5	Loss of form	
2	5-10	Loose stools	Hemoccult +
3	10-20	Watery Diarrhea	
4	>20	No feces produced	Gross bleading

Table S2. The Lichtiger Colitis Activity Index (LCAI) before and after dietary intervention

	Score in Lichtiger Index for UC	
Variable	Case 1 before/ after	Case 2 before/after
Diarrhea (number of daily stools)	4/1	4/1
Nocturnal diarrhea	0/0	0/0
Visible blood in stool (% of movements)	2/0	3/0
Fecal incontinence	0/0	0/0
Abdominal pain or cramping	3/0	3/0
General well-being	3/0	5/1

Abdominal tenderness	3/0	3/0
Need for antidiarrheal drugs	1/0	1/0
Total score	16/1	19/2

The Lichtiger Colitis Activity Index (LCAI) is a clinical tool used to assess the severity of symptoms in patients with ulcerative colitis. The scale interprets cut-off points as follows: 0-2: disease in remission (inactive), < 10: response to treatment, ≥ 10: active disease and no response to treatment. In both cases, a score well above 10 was achieved before the dietary intervention, indicating active disease. After dietary intervention, remission occurred in both cases, as indicated by a score in the range of 0-2.

Table S3. In vitro toxicity OBG data

	Fluorescence intensity repetitions			% of control repetitions		
Group	1	2	3	1	2	3
Control	22812	12621	21627	100	100	100
OBG 100 μg/mL	20456	18156	12393	89.6721	143.8555	57.303371
OBG 200 μg/mL	16431	12205	18191	72.02788	96.70391	84.112452
OBG 400 μg/mL	19352	10716	11234	84.83254	84.90611	51.944329
